# Enhanced adsorption capacity of activated carbon over thermal oxidation treatment for methylene blue removal: kinetics, equilibrium, thermodynamic, and reusability studies†

**DOI:** 10.1039/d2ra06481b

**Published:** 2022-12-21

**Authors:** Irwan Kurnia, Surachai Karnjanakom, Irkham Irkham, Haryono Haryono, Yohanes Andre Situmorang, Antonius Indarto, Atiek Rostika Noviyanti, Yeni Wahyuni Hartati, Guoqing Guan

**Affiliations:** Department of Chemistry, Faculty of Mathematics and Natural Sciences, Universitas Padjadjaran Jl. Raya Bandung – Sumedang KM. 21 Jatinangor Sumedang 45363 Indonesia Irwan.kurnia@unpad.ac.id +62-22-7794391; Study Center of Natural Resources, Energy and Environmental Engineering, Universitas Padjadjaran Jl. Raya Bandung – Sumedang KM. 21 Jatinangor Sumedang 45363 Indonesia; Department of Chemistry, Faculty of Science, Rangsit University Pathumthani 1200 Thailand; Department of Bioenergy Engineering and Chemurgy, Institut Teknologi Bandung Jl. Let. Jen. Purn. Dr. (HC). Mashudi No. 1 Sumedang 45363 Indonesia; Department of Chemical Engineering, Institut Teknologi Bandung Jl. Ganesha 10 Bandung 40132 Indonesia; Institute of Regional Innovation, Hirosaki University 3-Bunkyocho Hirosaki 036-8561 Japan

## Abstract

Activated carbon (AC) is an effective and inexpensive adsorbent material for dye removal, but it cannot always be used repeatedly. Furthermore, the adsorbed dyes with toxicity usually remain on its surface. In this study, a thermal air oxidation process was used to modify the surface of AC and decompose adsorbed methylene blue (MB). The behavior of this process on spent AC was investigated using TGA-DTA, while the degradation of MB before and after the regeneration process was analyzed using a carbon, hydrogen, nitrogen, sulfur (CHNS) analyzer. It was discovered that thermal air oxidation could promote the formation of oxygenated functional groups on AC produced from steam-activated carbon coconut shell (SACCS), which when treated at 350 °C (denoted as SACCS-350), demonstrated an adsorption capacity 2.8 times higher than the non-air-oxidized AC (SACCS). The key parameters for the MB adsorption of SACCS and SACCS-350, such as kinetics, equilibrium, and thermodynamics, were compared. Moreover, the SACCS-350 could be reused at least 3 times for the adsorption of MB. Based on these results, thermal air oxidation treatment could successfully improve the adsorption performance of AC and regenerate spent AC through a reasonable and environmentally friendly process compared to other regeneration methods.

## Abstract 2

Thermal air oxidation enhances the adsorption capacity of steam-activated carbon coconut shell (SACCS) on MB removal and recovers the adsorption ability of the spent SACCS treated at 350 °C.

## Graphical abstract

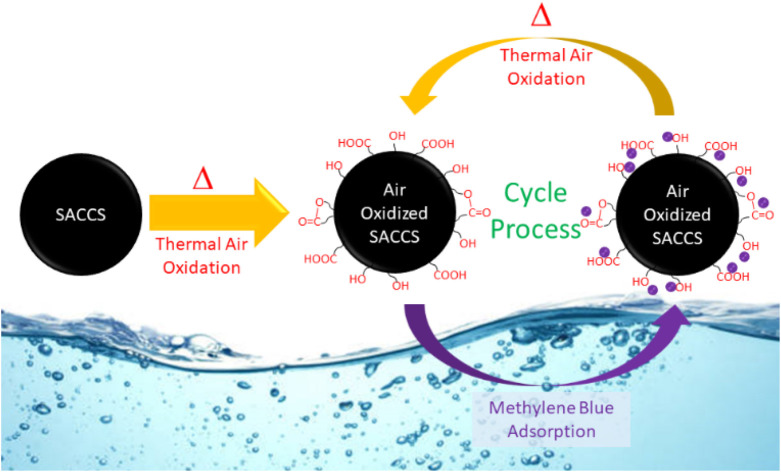

## Introduction

Wastes from the textile industry have led to severe environmental problems worldwide as dyes with high intensity are difficult to decompose through only natural biodegradation processes.^1^ It has been reported that about 10–15% of synthetic dyes are released into the environment through textile wastes^2^ and have a serious potential to disrupt the lives of aquatic organisms.^3^ However, the most widely used cationic synthetic dyes, such as methylene blue (MB), can also negatively impact humans and are more toxic than anionic synthetic dyes.^4^ Therefore, there is an urgent need to develop efficient methods for removing these wastes to ensure a sustainable ecology.

Several methods have been used to remove synthetic dyes, such as biodegradation, photocatalytic degradation, oxidation processes, membrane filtration, and adsorption.^5,6^ Among these, adsorption using activated carbon (AC) is the most popular because of its effectiveness and low cost.^7^ To enhance the adsorption capacity of AC for removing toxic contaminants, various physical, chemical, and biological treatment techniques have been applied,^8^ in which the generation of surface functional groups always plays a significant role in the adsorption process. It has been discovered that the chemical modification of AC, including by acid or alkali treatment, can greatly improve the efficiency of dye and heavy metals removal compared to other treatment methods.^9,10^ However, chemical treatment using highly corrosive acid or alkaline hydroxides produces a large amount of secondary wastewater in the post-treatment process. In response to such a problem, a simple thermal air oxidation method has been developed, in which oxygenated functional groups can be generated on the surface of AC, which is beneficial for the adsorption performance.^11,12^ In particular, it provides an environmentally friendly and low-cost means of enhancing the adsorption capacity of AC toward contaminant removal.

Nevertheless, the utilization of AC has a limitation in its repeated use for dye adsorption.^13^ In addition, the toxicity of the adsorbed dyes on the AC is a problem as these cannot decompose and reduce the toxicity.^14^ The regeneration processes applied for the spent AC from MB adsorption includes electrochemical,^15^ microwave,^16^ high-temperature treatment under a special atmosphere (N_2_,^17^ steam,^18^ or CO_2_ (ref. 19)), and thermal oxidation treatment.^19^ Among these, the most feasible and widely accepted for industrial applications is thermal air oxidation regeneration. However, there is no report on the behavior of this method during the regeneration process of spent AC and no details confirming that all the MB molecules are decomposed on AC. Therefore, it is necessary to investigate the thermal oxidation on spent AC by using a thermogravimetric analyzer coupled with a differential thermal analyzer (TGA-DTA), and to clarify the degradation of MB before and after the regeneration process utilizing a carbon, hydrogen, nitrogen, sulfur (CHNS) analyzer. In addition, reusability of the thermal air-regenerated adsorbent was observed for at least three cycles, whereas it was previously reported that only one cycle was possible.^19^

In this study, a thermal air oxidation process was applied to regenerate the spent AC to recover the adsorption performance and a pre-treatment process could increase the adsorption capacity. Meanwhile, this process was expected to be able to produce more oxygenated functional groups on the regenerated AC to enhance the adsorption capacity, as reported in the literature.^11^ The physicochemical properties of the modified adsorbent were characterized by Boehm titration and nitrogen adsorption measurements. Furthermore, the key parameters, such as kinetics, equilibrium, and thermodynamics, for MB adsorption were investigated.

## Experimental

### Modification of the AC surface by thermal air oxidation treatment

This study used commercial AC, such as Haycarb AKO 8 × 30 (Haycarb PLC, Sri Lanka), produced from steam-activated carbon coconut shell (SACCS),^20^ as a starting material for the adsorbent. First, the SACCS was ground and sieved to obtain a particle size of 0.1–0.3 mm, after which the surface of the SACCS was modified by thermal air oxidation treatment. Briefly, 1 g of SACCS was spread out on a porcelain ceramic crucible with a dimension of 120 × 60 mm, and then put in to a muffle furnace (Nabertherm B180, Nabertherm GmbH, Germany), and heated at a heating rate of 5 °C min^−1^ to the target final temperature (*i.e.*, 300 °C, 325 °C, 350 °C, 375 °C, and 400 °C, respectively), and then maintained at this final temperature for 1 h. Afterward, the sample was cooled to room temperature, and the obtained air-oxidized SACCS was denoted as SACCS-*X*, where *X* represents the target temperature.

### Characterizations

The yield from the thermal air oxidation treatment of the SACCS was calculated as the percentage of the mass of air-oxidized SACCS (SACCS-*X*) relative to the raw material. The amount of weakly acid functional groups in the SACCS-*X* was characterized by Boehm's titration method with different degrees of 0.01 N bases (NaOH, NaHCO_3_, and Na_2_CO_3_).^21^ Furthermore, the BET surface area and pore-size distribution of the adsorbent were determined through nitrogen adsorption measurements at 77 K using a surface area and pore-size analyzer (NOVA 4200e, Quantachrome Instrument Corp., USA). Degassing treatment was applied at 150 °C and 0.1 mbar before the nitrogen adsorption measurements. The surface areas of the SACCS and SACCS-*X* were determined in the range of *P*/*P*_0_ = 0.01–0.30. Additionally, elemental analysis of the adsorbent was performed with a CHNS analyzer (Vario EL Cube, Elementar Analysensysteme GmbH, Germany). The thermal air oxidation behavior on both the fresh and spent SACCS and SACCS-350 was characterized by TGA-DTA (STA200RV, Hitachi Co., Ltd., Japan).

### Adsorption experiment

The adsorption of methylene blue (MB) (Merck Millipore Corp., Singapore) on the SACCS and SACCS-350 were observed by adding 15 mg of adsorbent and 25 mL of MB solution (100 mg L^−1^) into a 50 mL centrifugation tube. The adsorption experiments were conducted on a multi-plate vortex (Joan Lab Equipment Co., Ltd., China) in an incubator chamber for 3 h with a shaker speed and temperature of 500 rpm and 30 °C, respectively. After reaching equilibrium, the suspension was centrifuged, and the solution was analyzed by UV-Vis spectrophotometer (Genesys 10S UV-Vis, Thermo Scientific Corp., USA) at a wavelength of 664 nm. The adsorption of each batch was performed at least 2 times, and the following equation was used to calculate the amount of adsorbed MB at equilibrium (*q*_e_, mg g^−1^):1
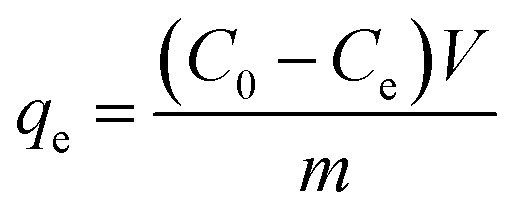
where *C*_e_ is the equilibrium concentration of the adsorbate (mg L^−1^), *C*_0_ is the initial concentration of the adsorbate (mg L^−1^), *V* is the volume of solution (L), and *m* is the mass of the adsorbent (g).

### Adsorption kinetics studies

The study of the kinetics model for MB adsorption on the SACCS or SACCS-350 was conducted in a similar way as the adsorption experiments in the time range of 10–360 min. The adsorption kinetics was determined with pseudo-first-order (PFO), pseudo-second-order (PSO), and intraparticle diffusion Weber–Morris (ID-WM) models. Table S1 (eqn (S1)–(S3))† shows the list of the mathematical equations for the kinetics models.

### Adsorption isotherm studies

The study of the isotherm model for MB adsorption on the SACCS or SACCS-350 was conducted as described in the Adsorption experiment section, with different initial concentrations of MB (25, 50, 100, 200, 300, and 400 mg L^−1^) for 3 h. The Langmuir, Freundlich, Redlich–Peterson, and Temkin models were used for the adsorption isotherm studies. Table S1 (eqn (S4)–(S7))† presents the list of the mathematical equations in the isotherm models.

### Determination of the thermodynamic parameters

The thermodynamic parameters for MB adsorption on the SACCS or SACCS-350 were determined as described in the Adsorption experiment section, with different temperatures of 30 °C, 40 °C, 50 °C, and 55 °C for 3 h.

### Normalized adsorption efficiency (NAE)

The normalized ratio of the adsorption efficiency was used to describe the visibility of the yield of SACCS-*X* toward the adsorption performance of SACCS-*X* compared to the starting material (SACCS). Therefore, the following was used to calculate the NAE:2
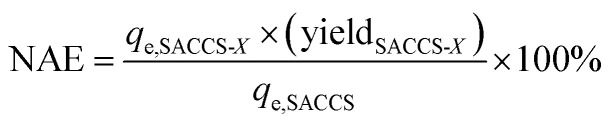
where *q*_e,SACCS_ and *q*_e,SACCS-*X*_ are the amount of adsorbed MB on SACCS and SACCS-*X* at equilibrium (mg g^−1^), respectively, and yield_SACCS-*X*_ is the yield of SACCS-*X* after thermal air oxidation treatment (%).

## Results and discussion

### Characterizations of SACCS and SACCS-*X*

In this study, steam-activated carbon coconut shell (SACCS) was used rather than chemical activated carbon in order to avoid the chemical impurities from the activation process, which would otherwise contribute to the MB adsorption. In the preliminary step, the temperature effect on the thermal air oxidation of SACCS toward the adsorption performance was studied in the temperature range of 300–400 °C for 1 h. Fig. S1† shows that the adsorbent yield decreased when the treatment temperature increased. TGA-DTA analysis was used to monitor the thermal air oxidation process of SACCS (Fig. S2a†), showing that the mass profile (TG) started to decrease from 150 °C and simultaneously the DTA profile exothermically increased, which corresponded to the oxidation process of SACCS (Fig. S2a†). Among those obtained at different temperatures through air oxidation, SACCS-400 showed the highest mass loss of 89.6 wt% due to the overoxidation (Fig. S1†). Therefore, it was not considered for the adsorption of MB due to the ineffective use of this adsorbent with a mass loss of more than 50 wt%.

**Fig. 1:**
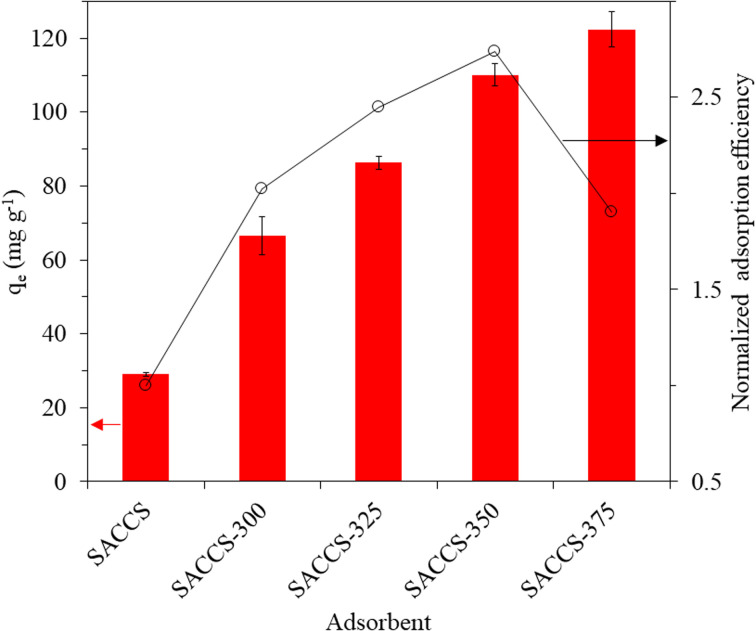
Adsorption efficiency (*q*_e_) and normalized adsorption efficiency from SACCS and SACCS-*X*. Adsorbent = 15 mg, MB = 100 mg L^−1^, 500 rpm, 30 °C, 180 min.

The adsorption efficiencies (*q*_e_) of SACCS and SACCS-*X* were evaluated for MB adsorption at 30 °C for 3 h. As summarized in Fig. 1, the adsorption efficiency of the SACCS-*X* increased with the elevation of the thermal air oxidation temperature. Herein, the enhanced performance resulted from the enriched total oxygenated functional groups (phenolic, lactonic, and carboxylic groups) on the surface of SACCS-*X*, as confirmed by Boehm titration, as shown in Table 1 and Fig. S3.† This was because they could improve the adsorption performance of the adsorbent due to electrostatic and hydrogen bond interactions.^11,22,23^ Meanwhile, the normalized adsorption efficiency (NAE) (eqn (2), Fig. 1) was further used to evaluate the adsorption performance. SACCS-375 showed the highest adsorption capacity, corresponding to the greatest amount of carboxylic groups with the lowest adsorption energy value compared to phenolic and lactonic groups.^24^ However, despite having the highest adsorption capacity, the NAE of SACCS-375 was lower than that of SACCS-350, which should be related to other surface properties. Table 1 shows that the surface area of SACCS increased from 467.9 to 688.0 m^2^ g^−1^ with the rise in air oxidation temperature until 350 °C, but then decreased to 621.8 m^2^ g^−1^ at 375 °C. A similar variation pattern also occurred on the cumulative pore volumes of SACCS-*X*. As a result, since SACCS-350 had a higher surface area and a larger cumulative pore volume, more MB molecules should be adsorbed on its surface,^22,25,26^ corresponding to the pore-size distribution, as shown in Fig. S4.† The increase in oxidation temperature resulted in the expansion of the SACCS half-pore width from 9–11 Å to 12–21 Å. However, treatment at over 350 °C led to the collapse of the adsorbent pore properties due to overoxidation, as indicated by the decreasing surface area and cumulative pore size of SACCS-375. These results also proved that SACCS-350 should have a higher normalized adsorption efficiency than SACCS-375. Therefore, SACCS-350 was selected as the optimum SACCS-*X* absorbent for the further equilibrium, kinetic, thermodynamic, and reusability studies on MB adsorption.

**Table 1:** Surface characteristics of SACCS and SACCS-*X*

Adsorbent	*S*_BET_ a (m^2^ g^−1^)	Cumulative pore volume^b^ (cm^3^ g^−1^)	Total oxygenated functional groups^c^ (μmol g^−1^)	Amount of each oxygenated functional group^c^		
				Phenolic groups (μmol g^−1^)	Lactonic groups (μmol g^−1^)	Carboxylic groups (μmol g^−1^)
SACCS	467.91	0.274	557.77	249.62	228.63	79.52
SACCS-300	571.57	0.341	668.66	168.66	241.55	258.45
SACCS-325	615.39	0.359	896.41	312.26	295.31	288.84
SACCS-350	688.02	0.408	1185.26	520.58	208.33	456.35
SACCS-375	621.77	0.370	1257.49	516.38	255.96	485.15

a *S*_BET_ was calculated using the N_2_ isotherm data from the multiple point method within a *P*/*P*_0_ lower than 0.30.

b DFT method cumulative pore volume.

c Determined by Boehm's titration method.

### Adsorption kinetics

**Fig. 2:**
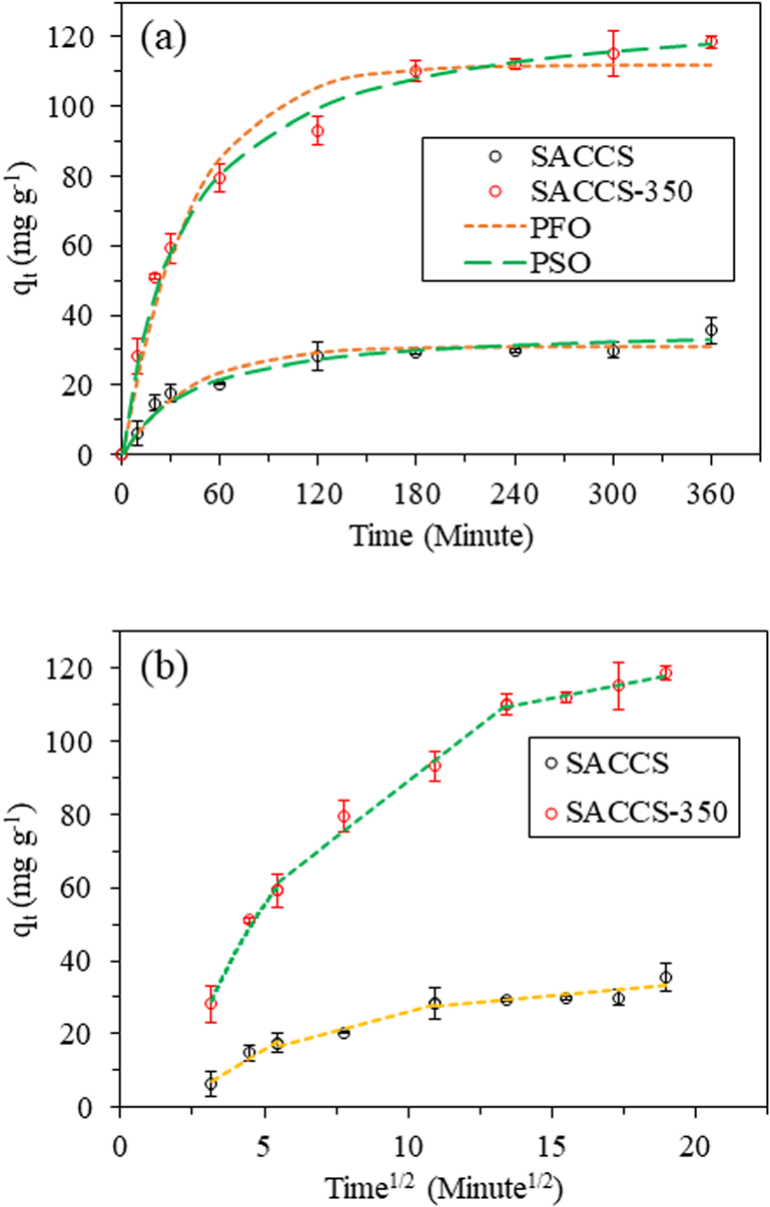
Adsorption kinetics of MB on SACCS and SACCS-350. (a) PFO and PSO kinetics models and (b) intraparticle diffusion of the Weber–Morris kinetics model. Adsorbent = 15 mg, MB = 100 mg L^−1^, 500 rpm, 30 °C.

The adsorption kinetics was used to describe adsorbate removal on the adsorbent, which is beneficial to understand the mechanism of the adsorption process. This study applied the PFO and PSO models to fit the kinetics data to explain the adsorption process, as presented in Table S1, eqn (S1)–(S3).†Fig. 2a and Table 2 show the adsorption kinetics of MB on SACCS and SACCS-350, and it could be observed that the adsorption profiles of MB on both SACCS and SACCS-350 increased with the extension of time. A rapid adsorption of MB occurred in the initial step, corresponding to a higher occupancy of adsorption sites for MB molecules. Subsequently, the rate of adsorption decreased at 60 min since a large amount of MB molecules were already attached to the adsorption sites, and then it reached equilibrium at 180 min. As indicated in Table 2, the PSO model fitted the adsorption kinetics for both SACCS and SACCS-350, with the values of *R*^2^ of 0.9794 and 0.9925, respectively. The calculated *q*_e_ values based on the PSO model were also close to the values for the experimental *q*_e_. These results indicated that MB adsorption on SACCS and SACCS-350 should occur in the chemisorption process, in which those oxygenated functional groups on SACCS and SACCS-350 should effectively contribute to anchoring the MB molecules.

**Table 2:** Kinetic parameters of the PFO, PSO, and intraparticle diffusion of the Weber–Morris (ID-WM) models for the adsorption of MB on SACCS and SACCS-350

Models	Parameters	SACCS	SACCS-350
Pseudo-first-order model (PFO)	*q*_e,exp_ (mg g^−1^)	29.05	110.08
	*q*_e_ (mg g^−1^)	13.51	34.31
	*k*_1_ (min^−1^)	0.0017	0.0070
	*R* ^2^	0.8876	0.9797
Pseudo-second-order model (PSO)	*q*_e_ (mg g^−1^)	36.65	129.75
	*k*_2_ (mg g^−1^ min^−1^)	0.0007	0.0002
	*R* ^2^	**0.9794**	**0.9925**
Intraparticle diffusion of Weber–Morris model (ID-WM)	*k*_ip1_ (mg g^−1^ min^1/2^)	4.8908	13.6524
	*k*_ip2_ (mg g^−1^ min^1/2^)	2.0189	6.1017
	*k*_ip3_ (mg g^−1^ min^1/2^)	0.7414	1.5490

The adsorption kinetics processes of SACCS and SACCS-350 occurred following the PSO model. However, the PSO model was unsuitable for the mechanistic analysis.^27^ As a result, the Weber–Morrison (ID-WM) model, which describes intraparticle diffusion, was applied to determine the reaction control step and the adsorption mechanism. Fig. 2b shows the multilinearity plots of *q*_*t*_*versus t*^1/2^, corresponding to the adsorptions of MB on both SACCS and SACCS-350 occurring in multiple steps of intraparticle diffusion.^28^ Herein, the parameters of the intraparticle diffusion model for SACCS-350 were 13.6524, 6.1017, and 1.5490 mg g^−1^ min^1/2^, respectively, which were higher than those of SACCS of 4.8908, 2.0189, and 0.7414 mg g^−1^ min^1/2^. These results indicated that the modification of SACCS with thermal air oxidation at 350 °C could increase the rate of MB adsorption, thereby improving the adsorption capacity. This indicated that the adsorption kinetics of MB on either SACCS or SACCS-350 should be controlled by chemisorption and intraparticle diffusion.

### Adsorption isotherms

**Fig. 3:**
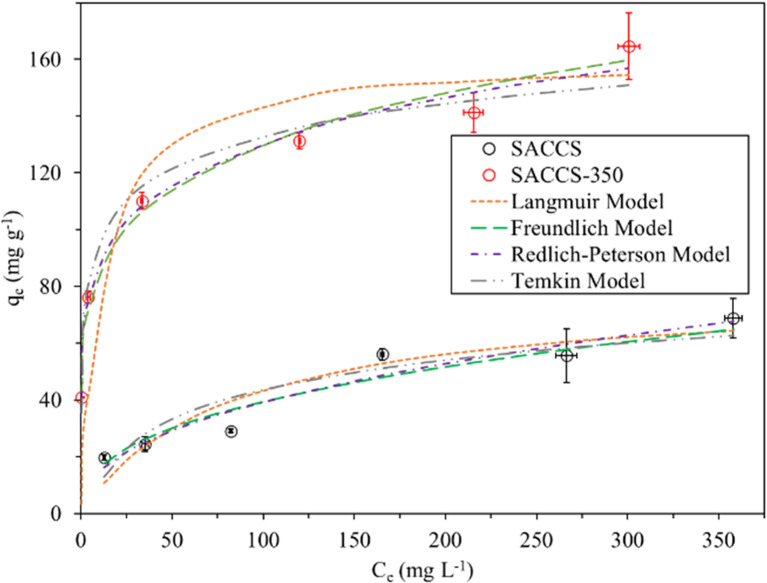
Adsorption isotherms of SACCS and SACCS-350. Adsorbent = 15 mg, 500 rpm, 30 °C, 180 min.

Isothermal adsorption is always used to determine the equilibrium relationship between the concentration of an adsorbate and the amount of accumulated adsorbate on the adsorbent. Here, the adsorption isotherm results were fitted with four models, namely the Langmuir, Freundlich, Redlich–Peterson, and Temkin models. The results of the fitting calculations are summarized in Fig. 3 and Table 3, while the linear and non-linear equations are listed in Table S1 (eqn (S4)–(S7)).† The order of the adsorption isotherm fitting models for SACCS was observed to be Redlich–Peterson > Langmuir > Freundlich > Temkin, while for SACCS-350, it was Freundlich > Langmuir > Temkin > Redlich–Peterson.

**Table 3:** Adsorption isotherm parameters of MB on SACCS and SACCS-350

Models	Parameters	SACCS	SACCS-350
Langmuir	*q*_max_ (mg g^−1^)	79.31	160.36
	*K*_L_ (L mg^−1^)	0.0121	0.0875
	*R* ^2^	0.9283	*0.9860*
Freundlich	*K*_F_ (mg g^−1^(L mg^−1^)^1/*n*^)	6.49	55.34
	1/*n*	0.3912	0.1856
	*R* ^2^	0.9264	**0.9908**
Redlich–Peterson (R–P)	*K*_RP_ (L g^−1^)	271.63	1218.91
	*α*_RP_ (L mg^−1^)	49.70	20.21
	*β*	0.5721	0.8324
	*R* ^2^	**0.9842**	0.8645
Temkin	*K*_T_ (L g^−1^)	0.1867	36.6021
	*H*_ads_ (J mol^−1^)	169.07	155.35
	*R* ^2^	0.8726	*0.9705*

The Langmuir isotherm assumes monolayer adsorption onto the homogeneous surface, where the adsorption sites have the same energy with no adsorbate interaction in the surface plane.^29,30^ However, the Freundlich isotherm is applied to describe the adsorption of heterogeneous surfaces with various affinities of adsorption sites.^31^ The results showed that SACCS-350 could be well-fitted to the Freundlich isotherm model with an *R*^2^ value of 0.9908, as shown in Table 3. This indicated the occurrence of different chemical interactions of the adsorbate with the heterogeneous oxygenated functional groups on the surface of SACCS-350. Meanwhile, the adsorption equilibrium of MB on SACCS was between the Langmuir and Freundlich models with *R*^2^ values of 0.9283 and 0.9264, respectively, as demonstrated in Table 3. Therefore, the Redlich–Peterson model could be applied to describe MB adsorption on the SACCS adsorbent, which includes features of the Langmuir and Freundlich isotherms, and represents the adsorption equilibrium over a broad range of concentrations of the adsorbate.^32^ The exponent, *β*, lay between 0 and 1. The Redlich–Peterson and Langmuir equations were the same when *β* = 1, but, at *β* = 0, it followed Henry's law. Since the Redlich–Peterson equation was related to SACCS with a *β* value of 0.5721 and *R*^2^ of 0.9842, it indicated that the behavior of the equilibrium isotherm of MB was between the Langmuir and Freundlich isotherms.

In addition, the Temkin isotherm model was also used to describe the MB adsorption equilibrium on SACCS and SACCS-350, in which the heat of adsorption of all the molecules on the layer was assumed to decrease linearly with the coverage due to the adsorbent–adsorbate interactions with a uniform distribution of binding energies.^33^ Herein, the positive values of *H*_ads_ for SACCS and SACCS-350 indicated that adsorption occurred in the endothermic process (Table 3). However, this model had a lower agreement for SACCS (*R*^2^ = 0.8726) and SACCS-350 (*R*^2^ = 0.9705) than the Redlich–Peterson model (*R*^2^ = 0.9842) for SACCS and Freundlich model (*R*^2^ = 0.9908) for SACCS-350, indicating that the variation in adsorption heat did not conform to the assumed linear decrease.

### Adsorption thermodynamics

The thermodynamic behavior was studied to determine the change of enthalpy (Δ*H*), entropy (Δ*S*), and Gibbs energy (Δ*G*) by considering the unit transfer of the adsorbate from the solution onto the liquid–solid interface. The thermodynamic parameters were calculated using the following equations:^34,35^3
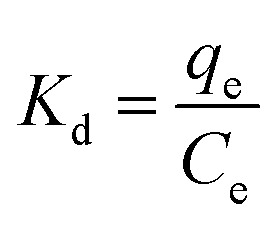

4 Δ*G* = −*RT* ln *K*_d_

5 Δ*G* = Δ*H* − *T*Δ*S*

6
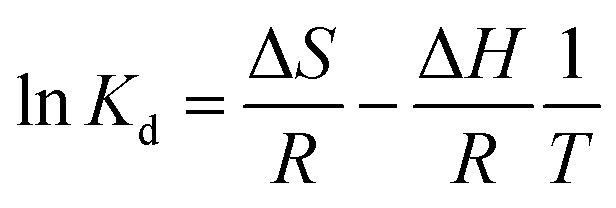
where *R* (8.314 J K^−1^ mol^−1^) is the universal gas constant, *T* (K) is the absolute solution temperature, and *K*_d_ is the distribution coefficient.

Table 4 shows the thermodynamic parameters for the adsorption of MB on SACCS and SACCS-350 in the temperature range of 30–55 °C (303–328 K), which were determined based on the results shown in Fig. S5.† It was observed that the increase in adsorption temperature resulted in a decrease in the Δ*G* value for both SACCS and SACCS-350. The positive value of Δ*G* for SACCS (Δ*G* = 2.62 to 0.48) implied that it was more difficult for MB adsorption to occur on the SACCS's surface. In contrast, the value of Δ*G* for SACCS-350 was negative, ranging from −2.99 to −6.01. This indicated that the adsorption process could occur naturally due to the thermal oxidation modification of the adsorbent surface.^36^ The positive Δ*S* values of both SACCS and SACCS-350 at 80.35 and 120.15 J K^−1^ mol^−1^, respectively, were attributed to the high disorder and randomness on the liquid–solid interface during MB adsorption. Meanwhile, the Δ*H* values of both SACCS and SACCS-350 were also positive, indicating that MB adsorption was an endothermic process.^37^

**Table 4:** Thermodynamics parameters of SACCS and SACCS-350 on MB adsorption. Adsorbent = 15 mg, MB = 100 mg L^−1^, 500 rpm, 180 min

Adsorbent	Δ*H* (kJ mol^−1^)	Δ*S* (J K^−1^ mol^−1^)	Δ*G* (kJ mol^−1^)				*R* ^2^
			303 K	313 K	323 K	328 K	
SACCS	26.99	80.35	2.62	1.80	1.23	0.48	0.9778
SACCS-350	33.39	120.15	−2.99	−4.24	−5.40	−6.01	0.9996

### Comparison of regeneration methods for spent AC

Table 5 compares the regeneration methods for spent activated carbon (AC) in MB adsorption. Among them, the microwave technique has the shortest time (0.05 h) to regenerate spent AC. The high equipment price for large-scale processes limits its implementation on the industrial scale. Furthermore, the adsorption capacity of regenerated AC decreases with the increasing adsorption–regeneration cycles due to the deterioration of the pore structure of AC.^16^

**Table 5:** Comparison of regeneration method for spent activated carbon in MB adsorption

No.	Activated carbon sources	SSA (m^2^ g^−1^)	*q*_max_ a (mg g^−1^)	*C*_0,max_ b (mg L^−1^)	Dosage^c^ (g L^−1^)	Time_max_^d^ (h)	Regeneration			Ref.
							Method	Time (h)	Cycles	
1	Bituminous coal (commercial)	862	371.75	600	1	480	Electrochemical (6 mA cm^−2^)	24	3	15
	PS: 0.55–0.75 mm									
	AM: n.a.									
2	Coconuts shells	1726	457	1000	1	2.5	Pyrolysis^e^ (635 °C)	0.5	1	17
	PS: 0.25–0.425 mm									
	AM: NaOH									
3	Spent coal (commercial)	1233	375.93	400	1	6	Steam (983 °C)	2.25	1	18
	PS: 2–3 mm									
	AM: n.a.									
4	Charcoal (commercial)	651 (SAC)^f^; 768 (Pyro-RAC) g; 730 (CO_2_-RAC)^h^	177 (SAC)^f^; 286 (Pyro-RAC)^g^; n.a. (CO_2_-RAC)^h^	1000	1	168	Thermal (350 °C); CO_2_ (900 °C)	1.5	1	19
	PS: 0.3 mm									
	AM: n.a.									
5	Coconut shells (commercial)	1131	154	1500	2	48	Microwave (200 Watt; 305 °C)	0.05	4	16
	PS: 1 mm									
	AM: steam									
6	Coconut shells (commercial)	467.91 (SACCS); 688.02 (SACCS-350)	79.31 (SACCS); 160.36 (SACCS-350)	400	0.6	3	Thermal (350 °C)	1	3	This work
	PS: 0.1–0.3 mm									
	AM: steam and thermal									

a Maximum monolayer adsorption capacity.

b Initial concentration of MB for *q*_max_ determination.

c Adsorbent dosage.

d Time for *q*_max_ determination.

e n N_2_ atmosphere.

f No treatment AC.

g Thermal regeneration AC.

h CO_2_ regeneration AC; SSA: specific surface area; PS: particle size; AM: activation method; n.a.: not available.

The high regeneration temperature of spent AC in N_2_, CO_2_, and steam conditions has also been studied, as presented in Table 5. However, there is no reusability data for more than 1 cycle. Also, the need for high-temperature treatment conditions impacts the high cost of the process.^17–19^

Unlike the above-mentioned regeneration processes for spent AC, the thermal air oxidation method does not require special equipment, and the treatment temperature is relatively low. Therefore, it represents a reasonable regeneration process for spent AC compared to the other approaches.

### Evaluation of the adsorbent reusability and feasibility of thermal air oxidation treatment

**Fig. 4:**
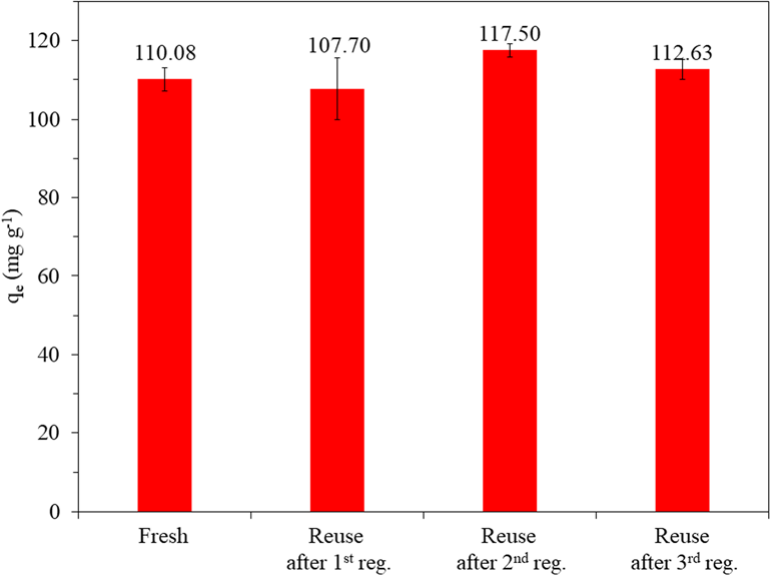
Reusability of SACCS-350 with a regeneration process at 350 °C. Adsorbent = 15 mg, MB = 100 mg L^−1^, 500 rpm, 30 °C, 180 min.

**Fig. 5:**
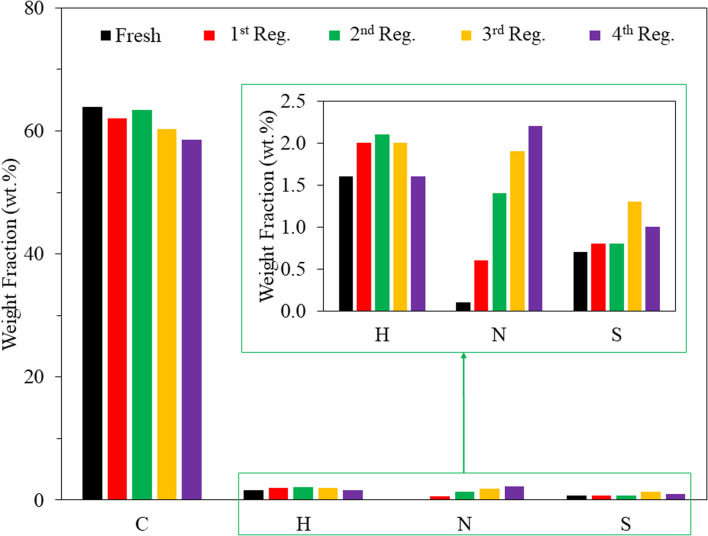
CHNS analysis of SACCS-350 before and after 4 times regeneration with the thermal air oxidation method at 350 °C for 1 h. The inset shows the enlargement of H, N, and S mass fraction elements.

Although the thermal oxidation method has been reported for the regeneration of spent AC,^19^ there is no information regarding the reusability of thermal air-regenerated adsorbents in more than one cycle. In this study, the reusability of the adsorbent was evaluated on SACCS-350 since it exhibited the highest adsorption capacity among all the SACCS and the SACCS-*X*. Also, studies were conducted without and with the regeneration process. Fig. S6† shows the reusability of SACCS-350 without regeneration, in which the adsorption capacity decreased from 110.08 to 44.0.6 mg g^−1^. This indicated that most of the adsorption sites of the fresh SACCS-350 were occupied with MB molecules in the first adsorption process. When the SACCS-350 was reused without regeneration, the adsorption capacity decreased due to the reduction in available adsorption sites. The thermal oxidation treatment of the spent SACCS-350 was performed to solve this problem. Fig. 4 shows that with the regeneration, the adsorption ability of the SACCS-350 could be completely recovered even after being reused and regenerated 3 times. To clarify the regeneration process, TGA-DTA analysis was used to observe the decomposition of the adsorbed MB. Fig. S2b† shows the differences between the TGA profiles of fresh and the regenerated SACCS-350. This indicated that the adsorbed MB could be completely decomposed with thermal oxidation treatment. Meanwhile, the decrease in the DTA profile from 200 °C to 500 °C should be related to the endothermic reaction due to the decomposition of MB. Despite the MB molecules decomposing, the nitrogen element remained and accumulated on the SACCS-350 even after four times of the regeneration process (Fig. 5). It should be noted that the accumulated nitrogen on the adsorbent after the regeneration process did not affect the adsorption capacity of the regenerated SACCS-350, hence, it could be reused for 3 times or more.

## Conclusions

Thermal air oxidation treatment was successfully used to increase the adsorption capacity of AC in MB removal and to regenerate spent AC. It was observed that SACCS-350 had the highest normalized adsorption efficiency (NAE). This was increased by 2.8 times compared to that of the non-treated SACCS. The adsorption kinetic process for SACCS and SACCS-350 followed the PSO model. Meanwhile, the equilibrium data of SACCS and SACCS-350 on MB adsorption showed good agreement with the Redlich–Peterson and Freundlich isotherm models, respectively. The thermodynamic study indicated that the thermal air oxidation treatment could make the endothermic adsorption processes more spontaneous. Furthermore, the thermal air oxidation treatment could recover the adsorption ability of the spent SACCS-350 at least 3 times. Therefore, this study can be expected to provide guidance to improve the adsorption effectiveness of activated carbon and to regenerate the spent AC so that it can be reused, resulting in a more environmentally friendly process and lower price.

## Author contributions

Irwan Kurnia: conceptualization, methodology, investigation, writing – original draft, project administration, supervision, resources. Surachai Karnjanakom: validation, writing – review & editing. Irkham Irkham: investigation, writing – review & editing. Haryono Haryono: investigation, writing – review & editing. Yohanes Andre Situmorang: investigation, writing – review & editing. Antonius Indarto: investigation, writing – review & editing. Atiek Rostika Noviyanti: investigation, writing – review & editing, resources. Yeni Wahyuni Hartati: investigation, writing – review & editing, resources. Guoqing Guan: validation, writing – review & editing.

## Conflicts of interest

There are no conflicts to declare.

## Supplementary Material

RA-013-D2RA06481B-s001
