# Supplementary material for: Enhanced adsorption capacity of activated carbon over thermal oxidation treatment for methylene blue removal: kinetics, equilibrium, thermodynamic, and reusability studies
Source: RSC Adv. 2022 Dec 21;13(1):220–7. doi: 10.1039/d2ra06481b (PMC9769092; doi:10.1039/d2ra06481b)
Supplement: RA-013-D2RA06481B-s001 [file RA-013-D2RA06481B-s001.pdf]

Electronic Supplementary Material (ESI) for RSC Advances.
This journal is © The Royal Society of Chemistry 2022

Supplementary Materials

Enhanced Adsorption Capacity of Activated Carbon over Thermal Oxidation Treatment for Methylene Blue Removal: Kinetic, Equilibrium, Thermodynamic, and Reusability Studies

*Irwan Kurnia**[a, b], *Surachai Karnjanakom*[c], *Irkham Irkham*[a, b], *Haryono Haryono*[a], *Yohanes Andre Situmorang*[d], *Antonius Indarto*[d, e], *Atiek Rostika Noviyanti*[a], *Yeni Wahyuni Hartati*[a], *Guoqing Guan*[f]

[a] *Department of Chemistry, Faculty of Mathematics and Natural Sciences, Universitas Padjadjaran, Jl. Raya Bandung – Sumedang KM. 21 Jatinangor, Sumedang 45363, Indonesia*

[b] *Study Center of Natural Resources, Energy and Environmental Engineering, Universitas Padjadjaran, Jl. Raya Bandung – Sumedang KM. 21 Jatinangor, Sumedang 45363, Indonesia*

[c] *Department of Chemistry, Faculty of Science, Rangsit University, Pathumthani 1200, Thailand*

[d] *Department of Bioenergy Engineering and Chemurgy, Institut Teknologi Bandung, Jl. Let. Jen. Purn. Dr. (HC). Mashudi No. 1, Sumedang 45363, Indonesia*

[e] *Department of Chemical Engineering, Institut Teknologi Bandung, Jl. Ganesha 10, Bandung 40132, Indonesia*

[f] *Institute of Regional Innovation, Hirosaki University, 3-Bunkyocho, Hirosaki 036-8561, Japan*

*Corresponding author.

E-mail address: irwan.kurnia@unpad.ac.id (I. Kurnia)

Table S1. Linear and non-linear equations of kinetics and isotherm models

No	Model	Non-linear equation	Linear equation
	Kinetic		
S1	PFO	$q_t = q_e(1 - exp^{-k_1 t})$	$\log(q_e - q_t) = \log q_e - \frac{k_1}{2.303}t$
S2	PSO	$q_t = \frac{k_2 {q_e}^2 t}{1 + k_2 q_e t}$	$\frac{t}{q_t} = \frac{1}{k_2 {q_e}^2} + \frac{1}{q_e}t$
S3	ID-WM	$q_t = q_e(1 - exp^{-k_{ip} t})$	
	Isotherm		
S4	Langmuir	$q_e = \frac{q_e K_L C_e}{1 + K_L C_e}$	$\frac{C_e}{q_e} = \frac{1}{q_{max} K_L} + \frac{1}{q_{max}} C_e$
S5	Freundlich	$q_e = K_F C_e^{1/n}$	$\log q_e = \log K_F + \frac{1}{n}\log C_e$
S6	Redlich-Peterson	$q_e = \frac{K_{RP} C_e}{1 + \alpha_{RP} {C_e}^{\beta}}$	$\ln\left(\frac{K_{RP}}{q_e} - 1\right) = \ln \alpha_{RP} + \beta ln C_e$
S7	Temkin	$q_e = \frac{RT}{H_{ads}} \ln(K_T C_e)$	$q_e = \frac{RT}{H_{ads}} \ln(K_T) + \frac{RT}{H_{ads}} \ln(C_e)$

q_e and q_t (mg g^{-1}) are the amounts of adsorbate at equilibrium and at time t, respectively; k_1 (min^{-1}) is are the pseudo-first-order rate constants and k_2 (mg g^{-1} min^{-1}) are the pseudo-first-order and the pseudo-second-order rate constants; k_{ip} (mg g^{-1} min$^{1/2}$) is the interparticle diffusion Weber-Morris rate constant; C_e (mg L^{-1}) is the equilibrium concentration of the adsorbate; q_{max} (mg g^{-1}) is the Langmuir maximum adsorption capacity of adsorbate per unit mass of adsorbent; K_L (L mg^{-1}) is the Langmuir constant related to the adsorption equilibrium; K_F (mg g^{-1} (L mg^{-1})$^{1/n}$) is the Freundlich constant related to the adsorption capacity; n (dimensionless) is the intensity of adsorption and constants incorporating the factors affecting the adsorption capacity; K_{RP} (L g^{-1})

and α_{RP} (L mg^{-1}) are Redlich-Peterson model constants; β is the exponent in between of 0 and 1; K_T (L/g) is Temkin constants; H_{ads} (J/mol) is enthalpy of adsorption.

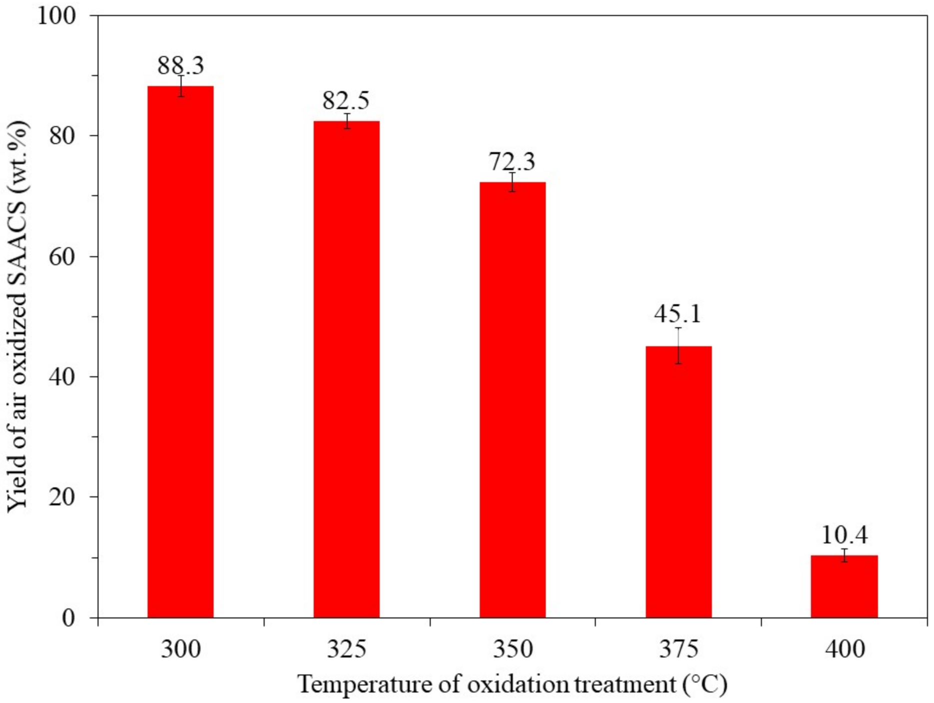

Fig. S1. The yield of air oxidized SACCS (SACCS-X) at different final temperatures.

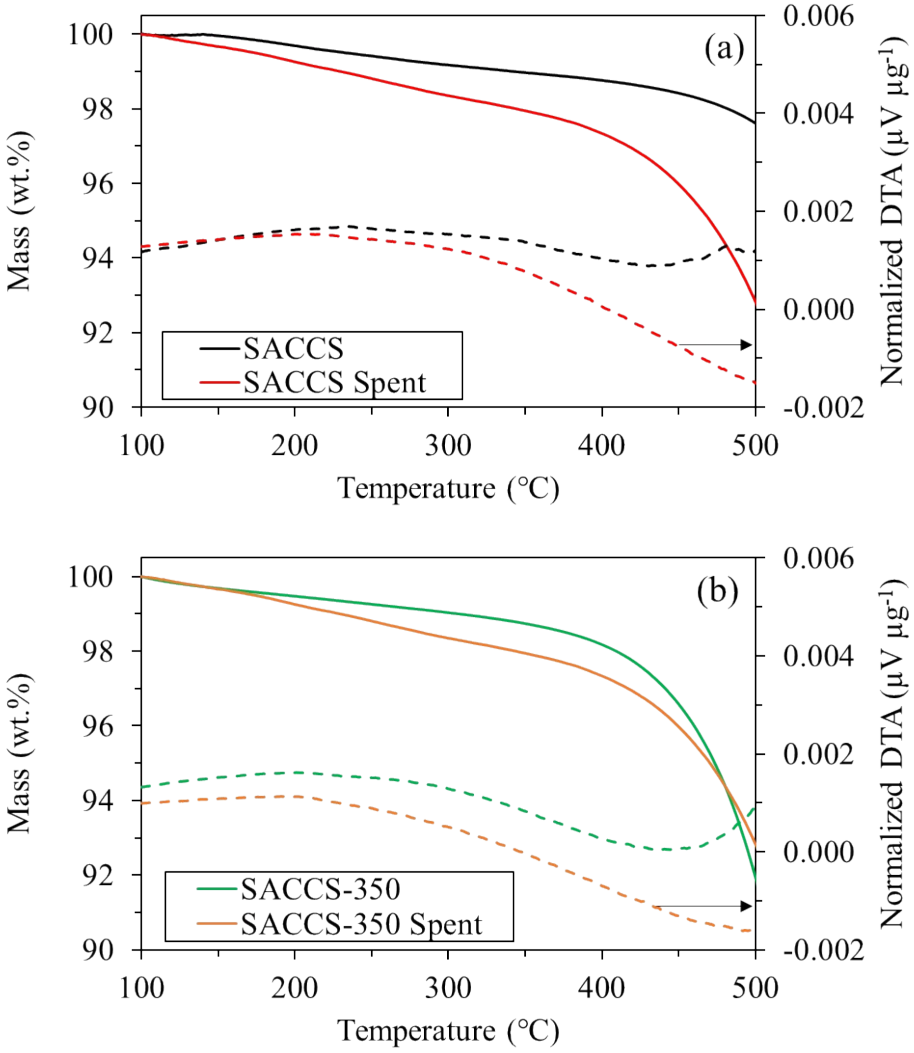

Fig. S2. TGA-DTA profiles of adsorbents. (a) SACCS and SACCS Spent; (b) SACCS-350 and SACCS-350 Spent.

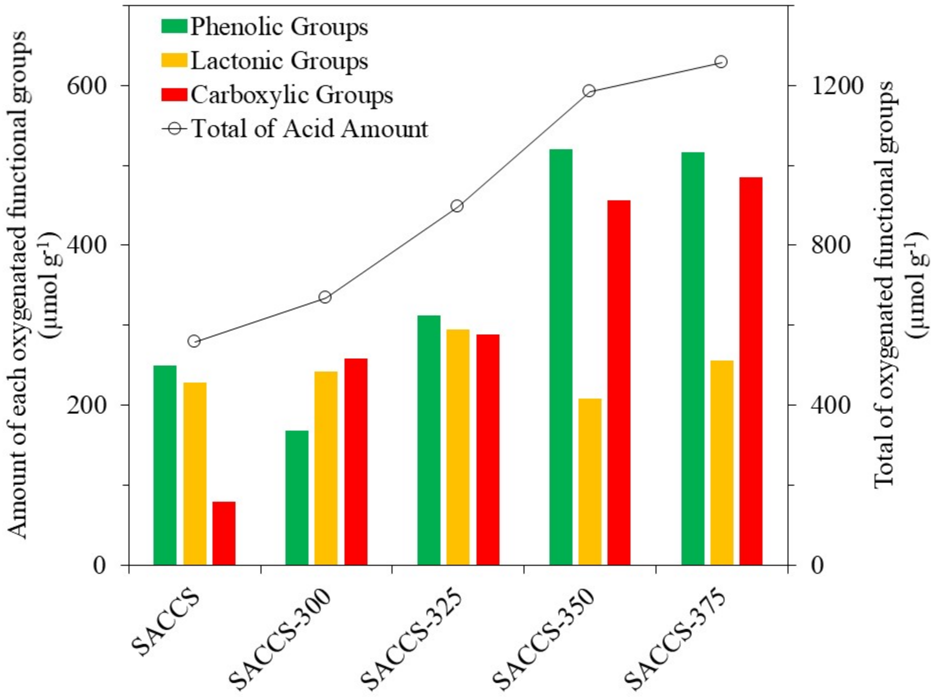

Fig. S3. The amounts of each oxygenated functional groups on SACCS and air oxidized SACCS-X.

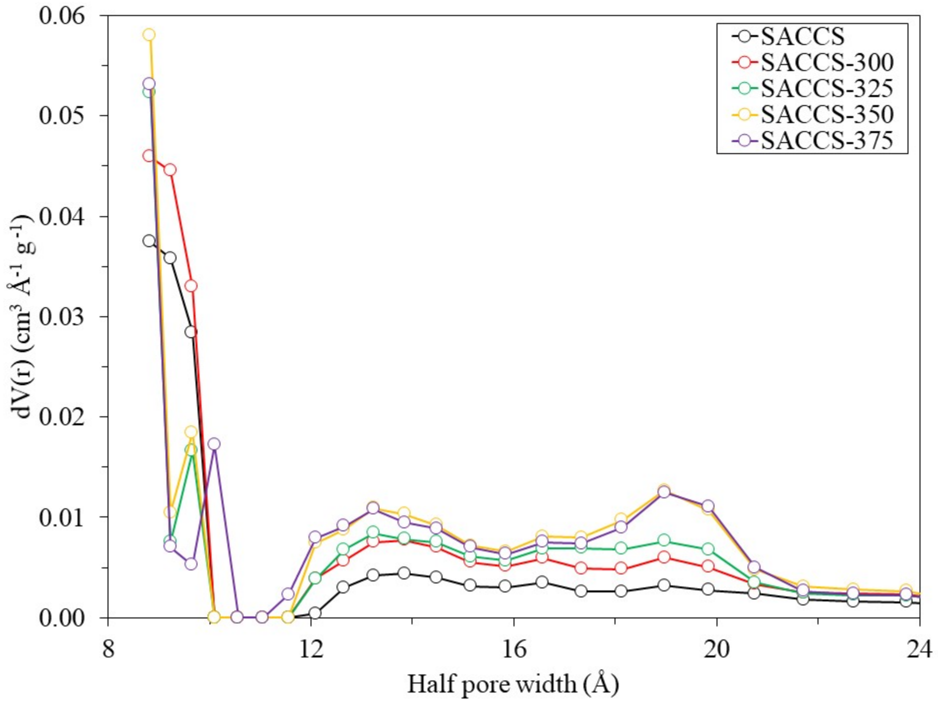

Fig. S4. Pore size distributions of the SACCS and air oxidized SACCS-X.

Pore size distribution was determined by NLDFT with slit pore model for carbon materials. The determination models of N_2 adsorption isotherms were measured at 77 K in the range of relative pressure of $P/P_0 = 0.01–1.00$.

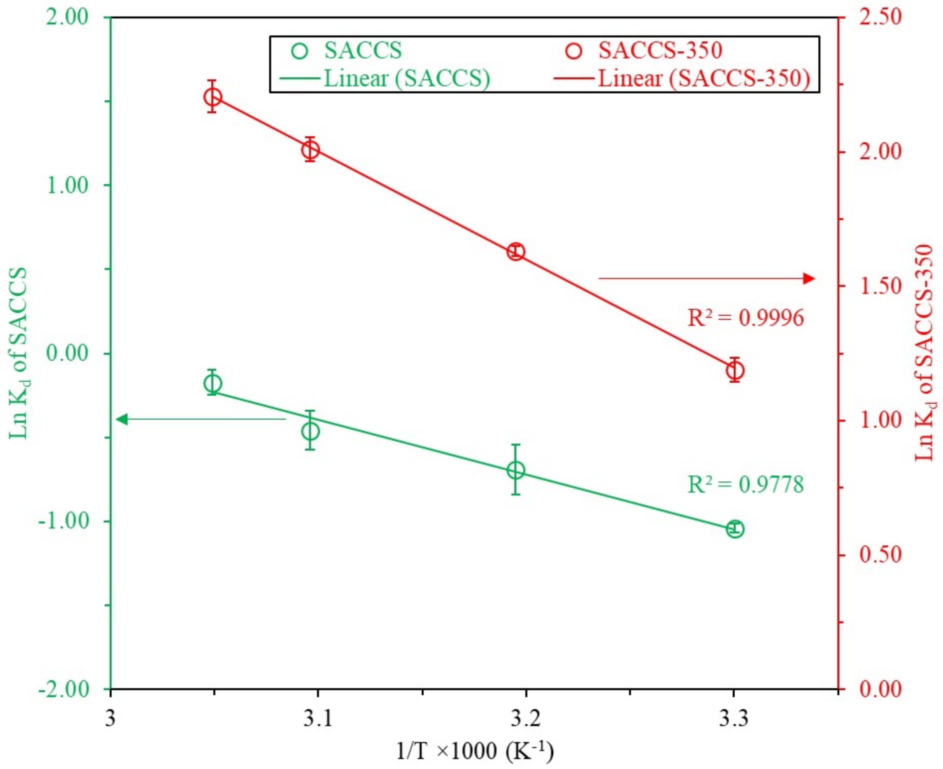

Fig. S5. Ln K_d vs 1000 T^{-1} plot for thermodynamic parameter determinations of SACCS and SACCS-350. Adsorbent = 15 mg, MB = 100 mg L^{-1}, 500 rpm, 180 min.

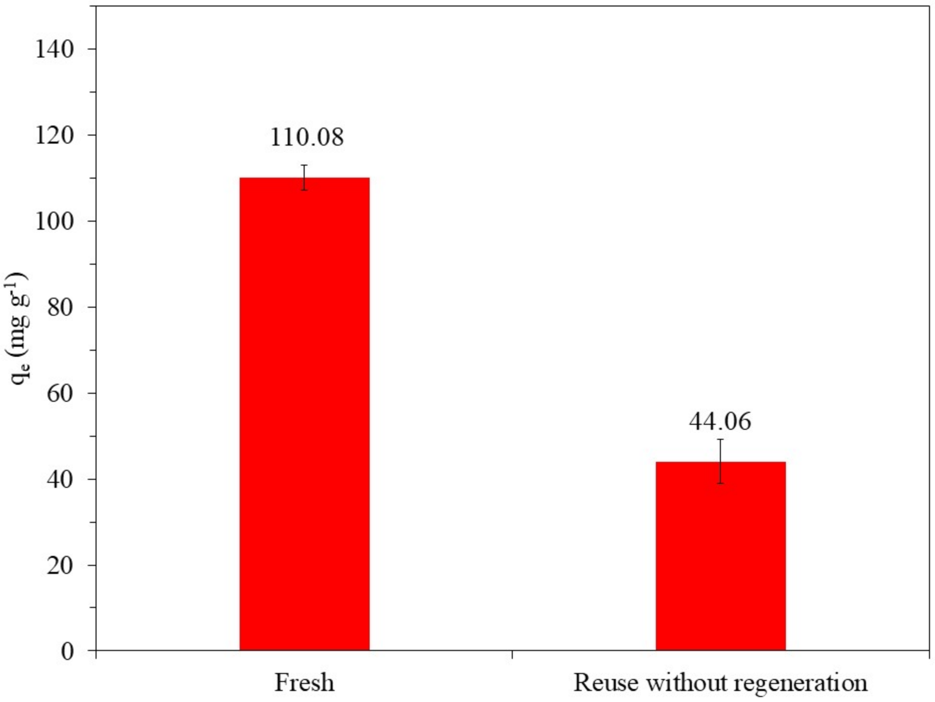

Fig. S6. Reusability of SACCS-350 without a regeneration process. Adsorbent = 15 mg, MB = 100 mg L^{-1}, 500 rpm, 30°C, 180 min.
